# Pathogen-on-a-Chip: Impedance-Based Detection of Biofilm Formation of *Staphylococcus aureus* and *Staphylococcus epidermidis*

**DOI:** 10.3390/bios15090596

**Published:** 2025-09-10

**Authors:** Bengisu Yöney, Radka Obořilová, Karel Lacina, Zdeněk Farka, Petr Skládal

**Affiliations:** 1 Department of Biochemistry, Faculty of Science, Masaryk University, Kamenice 5, 625 00 Brno, Czech Republic; bengisuyoney@mail.muni.cz (B.Y.); radka.oborilova@ceitec.muni.cz (R.O.); karel.lacina@ceitec.muni.cz (K.L.); farka@mail.muni.cz (Z.F.); 2 Central European Institute of Technology, Masaryk University, Kamenice 5, 625 00 Brno, Czech Republic

**Keywords:** microbial biofilm, electrochemical impedance spectroscopy, atomic force microscopy, disposable printed circuit board gold electrode

## Abstract

Bacterial biofilms are complex microbial communities that contribute to the pathogenesis of chronic infections. Therefore, it is crucial to detect biofilm-associated infections in early stages as their delayed treatment becomes more complicated. Herein, we describe a label-free electrochemical impedance spectroscopy (EIS) method for detecting biofilm formation by *Staphylococcus aureus* and *Staphylococcus epidermidis*. Printed circuit board-based biamperometric gold electrodes were modified with poly-L-lysine to enhance bacterial attachment to the sensor surface. Formation and inhibition of biofilms were evaluated based on changes in charge transfer resistance (*R*_ct_). The control *R*_ct_ value increased by ~90 kΩ for *S. epidermidis* biofilm and by ~60 kΩ for *S. aureus* biofilms. Antibiotic-treated samples exhibited similar values to those using the control. In addition, biofilm formation was evaluated through optical microscopy using safranin staining, and the micrographs suggest significant biomass on the electrodes, whereas the control appeared clear. Atomic force microscopy was used to visualize the biofilm on the electrode surface, obtain cross-sectional profiles, and evaluate its roughness. The roughness parameters indicate that *S. aureus* forms a rougher biofilm than *S. epidermidis*, while *S. epidermidis* forms a more compact biofilm. These findings suggest that the optimized EIS-based method effectively monitors changes related to biofilms and serves as a promising tool for evaluation of new anti-biofilm agents, such as antibiotics, phages or antibodies.

## 1. Introduction

In the microbial world, over 99% of microorganisms naturally exist as aggregated populations within self-produced biopolymers known as biofilms, which promote collaboration through a complex network [1]. Biofilms are formed by microbial cells that adhere tightly to a surface and produce an extracellular matrix (ECM) containing extracellular polymeric substances (EPS) that mainly composed of complex biomolecules, such as exopolysaccharides, extracellular DNA, proteins, and lipids [2,3]. Biofilms can form on a wide range of abiotic and biotic surfaces where bacteria adhere through various physicochemical interactions [4,5]. These interactions include Lifshitz-van der Waals forces, electrostatic double-layer contributions, and acid-base interactions [6]. The initial attachment of bacteria to the surface is influenced by multiple factors, including surface conditioning, mass transport, surface charge, hydrophobicity, and roughness [7]. All these factors contribute to initiation of biofilm formation, which provides a protective environment for bacteria.

Biofilms contain persistent cells, a dormant subpopulation of bacteria that exhibit high tolerance to antibiotics, contributing to the chronic and recurrent nature of biofilm-associated infections [8,9,10]. Bacterial biofilms lead to significant complications due to the complexity of treatment, as biofilm formation contributes to antibiotic resistance and uncontrolled persistence [11,12]. Once a (bio)material or a device has been implanted to a body, bacteria compete with host tissue cells to adhere to its surface and form a biofilm [13]. *Staphylococcus aureus* and *Staphylococcus epidermidis* are among the species well-known for forming biofilm on medical devices, causing infections, such as prosthetic heart valve infection, catheter biofilm infections, and blood stream infections [14,15,16,17,18].

Standard methods for observing biofilm formation include microscopic techniques: light microscopy, phase-contrast microscopy, confocal laser scanning microscopy, atomic force microscopy (AFM), scanning electron microscopy, and transmission electron microscopy. Suitable biological approaches include quantitative polymerase chain reaction, crystal violet assay, Congo red agar assay and determination of colony-forming units; however, these options are considered expensive, tedious, and time-consuming [19,20,21].

To address these limitations, impedance microbiology has been applied as a powerful technique to detect and quantify bacteria, and impedance-based methods have been implemented as a transduction mechanism [22]. Electrochemical impedance spectroscopy (EIS) has been used to understand biofilm phenomena due to its sensitivity, non-destructive nature, and the ability to monitor biofilm dynamics in real time [22,23]. EIS provides insights into biofilm behavior by examining capacitive (Z_C_), inductive (Z_I_), and Warburg (Z_w_) components of impedance, solution resistance (*R*_s_), and charge transfer resistance (*R*_ct_) [24,25]. Although EIS offers real-time biofilm monitoring, continuous impedance measurement in the presence of a redox probe may alter bacterial growth dynamics [26]. Compared to optical systems, electrical impedance biosensors provide quantitative data while enabling miniaturization, multiplexing, automation, and integration in lab-on-chip devices, along with the unique ability to characterize electrophysiological properties of different bacterial phenotypes [27]. Owing to these advantages, EIS has been adopted for biofilm detection on various electrode types, such as gold, indium-tin-oxide, and platinum [28,29,30,31]. However, many of these platforms rely on microfluidic systems or custom-made flow cells, which can be technically demanding and cost intensive.

This work proposes a two-electrode system utilizing gold electrodes based on the economic printed circuit board process; a poly-L-lysine (PLL) coating served for surface modification and better adhesion of biofilms. Altogether, this simplifies the setup, reduces costs, and supports miniaturization. We developed a low-cost, simple, and label-free impedance-based biosensor to detect biofilm formation with required sensitivity. Gold electrodes were modified with PLL to promote bacterial attachment, and the formation of biofilm by either *Staphylococcus aureus* or *Staphylococcus epidermidis* was monitored. In addition to EIS, optical microscopy and AFM were used to visually confirm presence of biofilm and changes on the surface. The biofilm-forming behavior of the two strains was compared using electrochemical and microscopic data. Biofilm inhibition was also initially investigated by applying amoxicillin (AMO) as an antibiotic.

## 2. Materials and Methods

### 2.1. Chemicals and Reagents

PLL and AMO were purchased from Sigma-Aldrich (St. Louis, MO, USA). Tryptone soya broth (TSB) was obtained from Oxoid (UK). Potassium hexacyanoferrate(II) was from Penta (Prague, Czech Republic). Potassium hexacyanoferrate(III) was obtained from Roth (Prague, Czech Republic). Alumina slurry (0.05 μm) was from Buehler (Lake Bluff, IL, USA). Safranin was purchased from Lachema (Brno, Czech Republic). All other chemicals were obtained from Sigma-Aldrich (USA) or Penta (Czech Republic). Tris-buffered saline (50 mM Tris, 150 mM NaCl, pH 7.5), and phosphate-buffered saline (10 mM NaH_2_PO_4_/Na_2_HPO_4_, 150 mM NaCl, pH 7.4; abbreviated as PBS) were prepared. Milli-Q water (Purelab Ultra, ELGA, High Wycombe, Buckinghamshire, UK) was used in this study.

### 2.2. Bacterial Strains and Cell Culture Conditions

The bacterial strains used in this study included *Staphylococcus aureus* RN4220 Δ*tarM* deletion mutant (*S. aureus*) and *Staphylococcus epidermidis* strain 1457-1000 (*S. epidermidis*), obtained from the Department of Genetics and Molecular Biology (Masaryk University). The bacterial cultures were stored in the freezer at −30 °C. They were inoculated into tryptic soy broth and incubated overnight at 37 °C in an incubator; the obtained optical densities at 600 nm were ~0.890 and ~0.870 for *S. aureus* and *S. epidermidis*, respectively. The cells were collected by centrifugation at 4600× *g* for 10 min and subsequently resuspended in TSB for further experiments.

### 2.3. Electrode Surface Modification

The printed circuit board electrodes were manufactured according to our design by the company Printed (www.printed.cz, accessed on 5 January 2025; Mělník, Czech Republic). EIS experiments were conducted using a two-electrode biamperometric system consisting of two similar gold electrodes (0.5 mm diameter). The electrodes were polished with 0.05 μm alumina slurry on a MicroCloth pad to remove impurities, rinsed with deionized water, followed by a 20 min incubation in basic Piranha solution (500 mM KOH, 3% H_2_O_2_) and thorough rinsing with deionized water; all steps were carried out at room temperature [32]. The electrodes were subsequently sterilized through ultraviolet (UV) light exposure for a duration of 30 min to prevent contamination. Finally, the electrodes were incubated with 30 µL of PLL (10 μg/mL) for 30 min and rinsed with deionized water [33].

### 2.4. S. aureus and S. epidermidis Biofilm Formation and Its Inhibition

*S. aureus* and *S. epidermidis* were immobilized by incubating the PLL-coated electrodes in each bacterial strain for either 1 h or 10 min. Afterwards, the electrodes were incubated at 37 °C for 24 h in TSB. For the antibiotic experiments, only the 10 min pre-incubation option was used; 5 mg/L AMO was added to TSB. For blank controls, the PLL-coated electrodes were directly transferred into either TSB or TSB containing AMO.

All experiments were performed in biological and technical triplicates for both bacterial strains. Sterile conditions were maintained throughout all procedures, and electrodes were handled using sterile forceps. After the incubation steps, the electrodes were collected and rinsed with distilled water and dried with compressed air for further analyses.

### 2.5. Electrochemical Impedance Spectroscopy Measurements

Electrochemical impedance spectroscopy was performed using the PalmSens4 potentiostat and PSTrace ver. 5.11 software system (Palm Instruments, Houten, The Netherlands). The measurements were conducted in 5 mL of 5 mM potassium hexacyanoferrate(II) and (III) solution in PBS. A frequency scan type was applied with 20 frequencies ranging from 100 kHz to 2 Hz, and the AC amplitude was set to 10 mV peak-to-peak, the DC component was 0 V [34]. Before all measurements, electrodes were rigorously washed with Tris buffer to remove the loosely bound planktonic bacteria from biofilms.

The acquired EIS data were analyzed using an equivalent circuit model consisting of a constant phase element CPE, *R*_s_, *R*_ct_, and *Z*_W_. In this study *R*_ct_ and *R*_s_ were evaluated. The fitting and analysis were performed using the software PSTrace.

### 2.6. Biofilm Evaluation Using Light Microscopy

Biofilm formation on gold electrode surfaces was examined using an BX41 light microscope (Olympus, Tokyo, Japan) with a Deep Sky Astro Camera 1.7 MP (Explore Scientific, Springdale, AR, USA). Magnifications and exposure times were 20×/450 ms, and 40×/1000 ms. Following incubation, the electrodes were gently washed with distilled water to remove planktonic cells without disrupting the biofilm attached to the surface. Subsequently, the samples were stained with safranin (0.5% *w*/*v*) at room temperature for 15 min to facilitate biofilm visualization [35]. Excessive stain was removed by rinsing the samples with PBS, followed by air-drying at room temperature.

### 2.7. AFM Characterization of Biofilms on Electrodes

Before AFM measurements, the biofilm on electrodes was rinsed with deionized water and air-dried. AFM was performed using the Dimension Icon (Bruker, Billerica, MA, USA) in PeakForce Tapping mode using the ScanAsyst-Air probe (Bruker) with a silicon tip on a nitride lever with a spring constant of 0.4 N/m. Atomic force micrographs (10 μm × 10 μm, resolution 512 × 512 pixels) were processed using the Gwyddion ver. 2.69 software (Czech Metrology Institute, Brno, Czech Republic) [36]. For each micrograph, roughness was measured in three different regions, each with a line of 9 μm.

### 2.8. Statistical Data Analysis

Triplicates were conducted in all experiments involving biological samples and measurements. Data analysis was conducted using Origin 2023 (OriginLab, Northampton, MA, USA). A scatter interval chart was used to represent the mean values along with standard errors (SE) to visualize the variability between the replicates. Statistical analysis was conducted using GraphPad Prism ver. 10 (GraphPad Software, Boston, MA, USA). Statistical significance was evaluated and is represented by asterisks: **** for *p* < 0.0001, *** for *p* < 0.001, ** for *p* < 0.01, and * for *p* < 0.05.

## 3. Results and Discussion

### 3.1. Electrochemical Evaluation of Biofilms

The electrode surface was firstly cleaned using an alkaline Piranha solution to remove impurities and organic substances and to introduce hydroxyl groups on the surface. Surface hydroxylation with its partial negative charge plays a crucial role in enhancing the attachment of PLL, the positive charges of which in turn help capturing both Gram-positive and Gram-negative bacteria via electrostatic interactions [37]. In this study, *S. aureus* and *S. epidermidis*, both Gram-positive bacteria, were successfully captured using this approach. Similarly, Marka et al. employed PLL-coated gold electrodes to promote cell attachment prior to impedance measurements which demonstrated the efficacy of PLL in facilitating initial cell adhesion on surfaces before further formation of biofilms [38].

**Figure 1:**
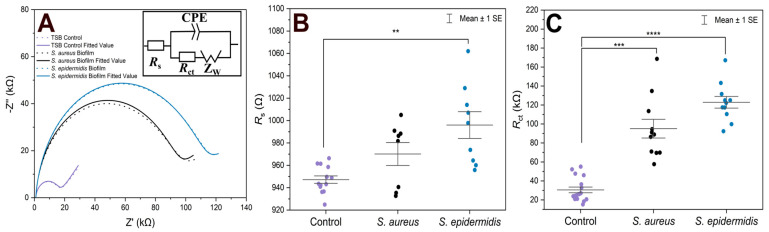
(**A**) Nyquist plots of [Fe(CN)_6_]^3−/4−^ at gold electrodes in PBS as supporting electrolyte after being incubated under different conditions for 24 h. The solid traces represent illustrative experimental data, while the dashed traces correspond to the fitted values obtained using the Randles equivalent circuit model consisting of solution resistance (*R*_s_), constant phase element (CPE, or Q), charge transfer resistance (*R*_ct_), and Warburg element (W). The results of fitting with alternative equivalent circuits are summarized in Table 1. (**B**) Scatter plot with error bars representing the distribution of *R*_s_ values obtained from equivalent circuit fitting for gold electrodes incubated in TSB. (**C**) *R*_ct_ values obtained from equivalent circuit fitting for gold electrodes incubated in TSB. Each point represents an independent measurement, and horizontal bars indicate mean ± standard error. Statistical significance is indicated by asterisks: **** for *p* < 0.0001, *** for *p* < 0.001, ** for *p* < 0.01.

For EIS experiments, PLL-coated electrodes were first incubated with *S. aureus*, *S. epidermidis*, or TSB (control) for 1 h, followed by their incubation in TSB nutrient medium at 37 °C for 24 h. Under such conditions, the formation of biofilms was expected [39]. The EIS procedure was employed to verify biofilm formation on both electrodes that were used in the electrochemical cell, and to compare two bacterial strains on the modified surfaces. In terms of biofilm detection, both *R*_s_ and *R*_ct_ were evaluated and compared (Figure 1). The Nyquist (imaginary impedance (*Z*″) versus real impedance (*Z*′)) plots of [Fe(CN)_6_]^3−/4−^ at *S. aureus*- and *S. epidermidis*-immobilized gold electrodes and the control electrode in PBS (7.4) are displayed in Figure 1A. In these Nyquist plots, the diameter of the semicircular feature in the high frequency region is used to estimate *R*_ct_, while the off set on the real impedance axis from the origin corresponds to *R*_s_. Based on the equivalent circuit displayed in the inset of Figure 1A, simulated Nyquist plots were also generated, and they are represented by dashed traces in Figure 1A. A plot of the *R*_s_ values (Figure 1B) of the three electrode systems in Figure 1B shows a statistically significant difference in *R*_s_ (*p* < 0.01) between the control and *S. epidermidis* biofilm. This is expected as *R*_s_ mostly corresponds to the properties of the surrounding medium. On the other hand, the *R*_ct_ values (Figure 1C), representing the charge transfer resistance of [Fe(CN)_6_]^3−/4−^ at the biofilm-coated electrodes, were able to clearly distinguish between biofilms and control groups. The other parameters are presented in Table 1, CPE is capacitance arising from the biofilm layer, the Helmholtz double layer and surface roughness of the electrodes, and *Z*_W_ is Warburg impedance or diffusion-limit interfacial resistance.

In the case of controls, the fluctuations in both *R*_s_ and *R*_ct_ combine variability of the electrode sizes and pretreatment procedures, plus some contribution of adsorbed species from the cultivation medium used also in the blank experiments. Additionally, in several cases presented in Figure 1B, the *R*_s_ values for the *S. aureus* biofilms did not change significantly compared to the controls. This can be expected as the surrounding solution was the same. In fact, the upper surface of biofilms is rather dynamic without precisely defined boundary; there are some loosely bound bacteria which can be released during the EIS procedure. On the other hand, the *R*_ct_ values (Figure 1C) clearly distinguished between biofilm and control groups. The *R*_ct_ values revealed that *S. epidermidis* exhibited ~120 kΩ increased value (an average) compared to the blank control, whereas *S. aureus* showed ~90 kΩ change with wider distribution range in *R*_ct_ values. Moreover, *S. epidermidis* biofilm formation showed higher statistical significance compared to the control (*p* < 0.0001) than *S. aureus* (*p* < 0.001).

Several other circuits were also considered for fitting the experimental traces from Figure 1C, the comparison of fits based on equivalent circuit parameters and the Chi-squared (χ^2^) sum of residuals is provided in Table 1.

All the selected models provided very similar *R*_s_ values around 1 kΩ. The *R*_s_([*R*_ct_*W*]*C*) model (the second line in Table 1) failed to evaluate the *R*_ct_ values for the electrodes with biofilms, the C results (high parameter deviations) unexpectedly decreased, though the increase in Warburg parameters *W* was reasonable. The *R*_s_(*R*_1_*C*_1_)(*R*_ct_*C*) model (the third line in Table 1) might seem appropriate, as we expect two layers—PLL and biofilm on the electrodes. However, the resulting *R* and *C* sets of results look rather fluctuating probably due to the failure of the fitting algorithm. The simplified *R*_s_(*R*_ct_*C*) and classic *R*_s_([*R*_ct_*W*]*Q*) Randles models (the first and last lines in Table 1, respectively) performed rather well and provided similar changes and even the absolute values for *R*_ct_. The trends in *C* and *Q* values were also similar though rather small increases for the biofilm-coated electrodes were obtained. This indicates that biofilm is not providing sufficient isolation of the electrode to modify its capacitance characteristics. Thus, relaying on the *R*_ct_ parameter seems more reasonable for evaluating biofilm formation and its changes. Some minor increases were identified also for the Warburg values, but with substantially higher relative deviations compared to *R*_ct_. The finally adopted classic Randles circuit (the last line in Table 1) provided the smallest sums of residuals, and it was used throughout for fitting the following EIS traces.

Previous studies confirm our results regarding *R*_ct,_ which can be sensitively monitored using EIS. For example, Oliver et al. investigated the monitoring of impedance during biofilm formation by *S. epidermidis* RP62A on gold electrodes and reported an increase in *R*_ct_ over time [40]. This suggests that *R*_ct_ is a sensitive indicator for detection of biofilm on the electrode surface.

### 3.2. Validation of Biofilm Formation on Electrodes

**Figure 2:**
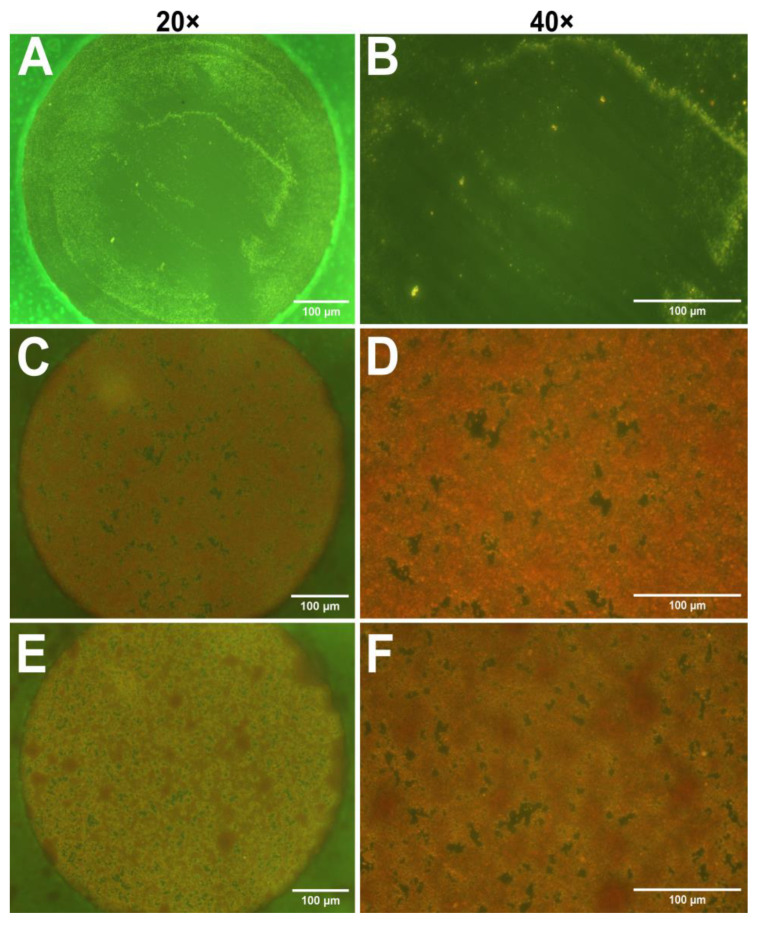
Optical micrographs of gold electrodes stained with safranin to visualize biofilm formation. (**A**,**B**) TSB control electrodes at 20× (**A**) and 40× (**B**) magnifications. (**C**,**D**) Electrodes incubated with *S. aureus*, magnification 20× (**C**) and 40× (**D**). (**E**,**F**) Electrodes incubated with *S. epidermidis*, magnifications 20× (**E**) and 40× (**F**). Scale bars: 100 µm.

After the impedance measurements suggested the presence of biofilms, optical microscopy was employed to visualize biofilm on the electrodes. In this study safranin dye was utilized, as it is often used for staining cell nuclei in histology [41]. However, Ommen et al. reported that safranin can also serve as a reliable staining agent for biofilm biomass quantitation [42]. Figure 2 presents micrographs of safranin-stained electrodes incubated with only TSB (control), *S. aureus*, and *S. epidermidis* at 20× and 40× magnifications. As expected, the control sample contains no bacteria and thus no visible dye retention; however, some surface impurities probably caused by the polishing step can be observed. The micrographs obtained from bacterium-incubated electrodes in Figure 2C–F show specific red coloration of safranin, indicating the presence of a biofilm on the electrodes. However, it is difficult to distinguish between the two strains. Due to the limitations of optical microscopy, it is essential to use a complementary technique, such as AFM.

AFM can be used to study whole biofilms or their components, such as the ECM, offering high resolution [43]. It also enables the observation of changes in the size of bacterial cells during growth and biofilm formation. In addition, height profiles and roughness parameters obtained from AFM provide insights into homogeneity and surface morphology of biofilms. Based on these benefits, AFM was employed in this study to investigate the biofilm structure in greater detail.

**Figure 3:**
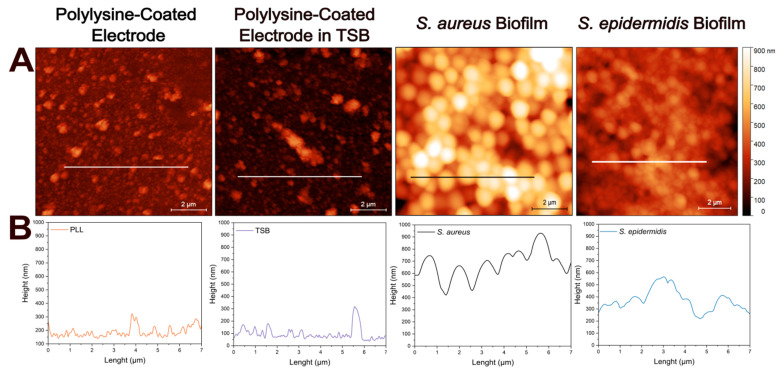
Atomic force microscopy analysis of electrode surfaces in the absence and in the presence of biofilms. (**A**) Height sensor microscopic analysis of: PLL-coated bare electrode, PLL-coated electrode incubated in TSB, PLL-coated electrode with *S. aureus* biofilm, and PLL-coated electrode with *S. epidermidis* biofilm. Surface topography reveals biomass accumulation in the presence of bacterial biofilms compared to control electrodes. (**B**) Representative height profiles corresponding to each condition along the marked 7 µm lines. Scale bar: 2 µm.

Figure 3A illustrates the atomic force micrographs of the control and biofilm-coated electrodes along with the corresponding cross-sectional profile lines (Figure 3B) for each sample. The atomic force micrographs based on height profiles demonstrate no significant surface differences between the PLL-coated electrode and the electrode after incubation in TSB at 37C for 24 h. The cross-sectional profile of bacteria lacking extracellular matrix reveals a smooth and continuous contour, corresponding to the spherical morphology of individual cells as in *S. aureus*. However, when extracellular matrix is present, the surface becomes markedly rougher and more irregular, displaying increased topographical complexity likely associated with biofilm-incorporated structural components as in *S. epidermidis*. Here, cross-sectional height profile is determined along 7 µm long lines to provide detailed morphologies of the samples.

The observed trend likely reflects fundamental differences in biofilm architecture and extracellular matrix composition between the two species. *S. epidermidis* is known to produce a denser and more cohesive biofilm, which can increase both the solution resistance (due to limited ionic mobility in the matrix) and the *R*_ct_ (due to hindered access to the electrode surface). This interpretation is supported by the atomic force micrographs, where *S. epidermidis* formed more uniform and confluent layers with a greater surface coverage (in the case of 1 h immobilization), in contrast to the patchier and irregular structure observed for *S. aureus*. The EIS fitting further supports this explanation, as the higher *R*_ct_ values correlate with more compact biofilms impeding electron transfer at the interface.

### 3.3. Inhibition of Biofilm Formation

In this part, the PLL-coated electrodes were incubated with either *S. aureus* or *S. epidermidis* for only 10 min prior to biofilm formation. This shorter incubation period was chosen to reduce the overall experimental time. We aimed to inhibit the biofilm development by applying 5 mg/L of the antibiotic AMO) and monitoring the resulting changes via EIS. The electrodes were incubated in a mixture of AMO and TSB medium, and after 24 h, EIS measurements were performed as previously, followed by AFM imaging.

**Figure 4:**
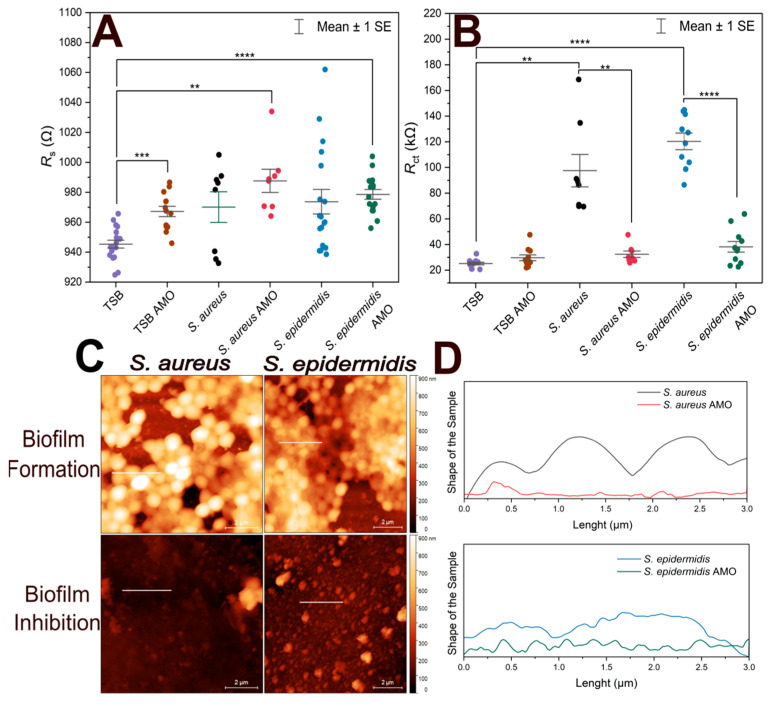
Comparison of short-term bacterial immobilization and biofilm inhibition on gold electrodes. (**A**) Scatter plot of *R*_s_ values for biofilm-forming electrodes, antibiotic-treated electrodes, TSB with antibiotics (TSB AMO), and TSB (control). (**B**) Scatter plot of *R*_ct_ values for biofilm-forming electrodes, antibiotic-treated electrodes, TSB with antibiotics (TSB AMO), and TSB (control). (**C**) AFM height sensor images of electrodes incubated with *S. aureus* and *S. epidermidis* in TSB (biofilm formation) and in TSB containing antibiotics (biofilm inhibition). Scale bar: 2 µm. Horizontal lines indicate the evaluated height profiles. (**D**) Height profiles along 3 µm long lines on each electrode surface, showing differences in vertical features between biofilm-forming and biofilm-inhibited conditions. The vertical axes extents correspond to 1 µm. Biological and technical replicates are represented, and horizontal bars indicate mean ± standard error. Statistical significance is indicated by asterisks: **** for *p* < 0.0001, *** for *p* < 0.001, ** for *p* < 0.01.

Figure 4 shows the variation of *R*_s_, *R*_ct_ from EIS experiments, and height profiles of electrodes coated by *S. aureus* and *S. epidermidis* from atomic force microscopic investigations.

To assess biofilm inhibition, both *R*_s_ and *R*_ct_ values were compared, as shown in Figure 4A. TSB control exhibited a statistically significant difference compared to TSB with AMO, *S. epidermidis* in AMO and TSB, and *S. aureus* in AMO and TSB. This may suggest that the presence of AMO in the medium for 24 h can slightly influence the solution resistance. On the other hand, biofilm formation itself did not result in a notable difference in *R*_s_ values.

EIS results obtained by fitting the data with the equivalent circuit model, along with the corresponding *R*_ct_ values, are presented in Figure 4B. *S. aureus* exhibited ~100 kΩ and more variable *R*_ct_ values compared to *S. epidermidis* (~120 kΩ), consistent with the previous 1-h immobilization results. Electrodes incubated with the AMO solution showed lower mean *R*_ct_ values, which were similar to the TSB + AMO control and the TSB control.

There was no statistically significant difference between 10 min and 1 h immobilization in terms of *R*_ct_ values (*p* > 0.05) for both *S. epidermidis* and *S. aureus* biofilms. This suggests that regardless of the initial incubation with bacteria, the biofilm formed on the electrode after incubation at optimum conditions is sufficient to cause an increase in *R*_ct_.

In Figure 4C, atomic force micrographs reveal that biofilm-forming electrodes contain not only biomass but also display empty areas; the coverage areas are quantified in Table 2 together with the maximum profile height parameters. It seems that over time, the vacant regions will be filled by ECM. The electrodes incubated with AMO display no visible biofilm formation. It can be seen that in the case of longer 1 h immobilization, *S. epidermidis* creates a biofilm with a larger surface area and from the maximum peak values we observe that this biofilm is lower, but we assume that it is therefore more compact than in the case of *S. aureus*, which forms rather less distributed, more fragmented structures.

The size of individual bacteria estimated from Figure 4C was 990 ± 110 nm and 760 ± 120 nm for the *S. aureus* and *S. epidermidis* biofilms, respectively (mean values ± standard deviations, *n* = 10). Notably, measuring bacterial size at the biofilm stage may not be entirely reliable: cells are continuously dividing, which can affect the estimations when capturing cells just before or just after division. Nevertheless, the measurements provide useful information for comparative purposes, showing that *S. epidermidis* is significantly smaller than *S. aureus* at this growth stage. The difference in bacterial sizes was confirmed as significant by a two-sample *t*-test (*p* = 0.00047).

The height profiles in Figure 4D indicate that biofilm-forming electrodes exhibit cell-like structures, whereas the AMO-samples show the electrode surface without any cell. In this experiment, cross-sectional height profile length is determined along 3 µm long lines to compare the differences between biofilm-forming electrodes and antibiotic-treated electrodes. Height profiles demonstrate that the *S. aureus* biofilm has preserved the shape of individual cells [44]. In contrast, *S. epidermidis* lacks the typical structure of individual bacterial cells because the cells are embedded in the EPS, which causes a compact profile.

**Figure 5:**
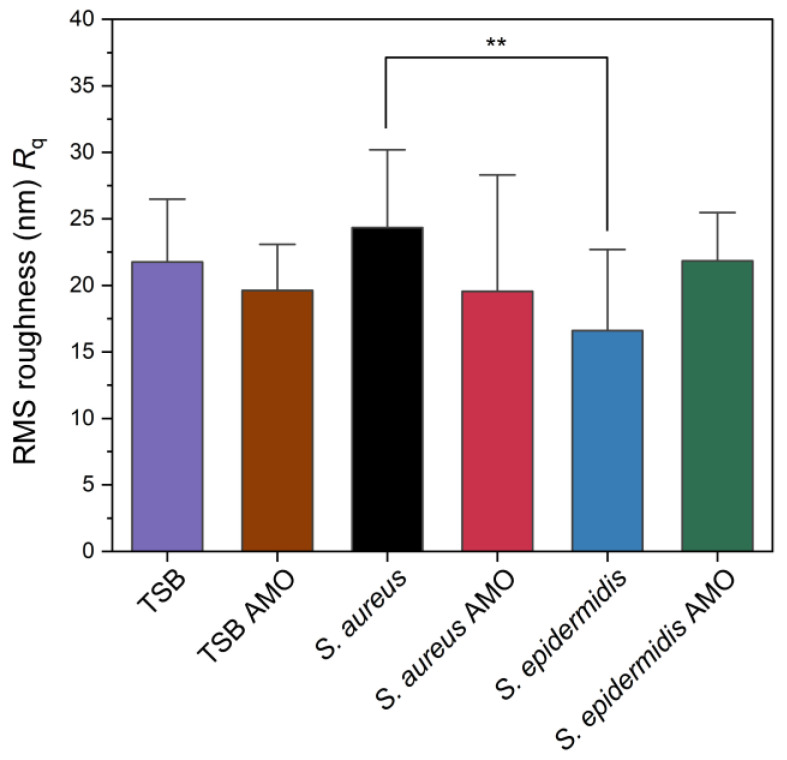
Root mean square (RMS) surface roughness (*R*_q_) values of control, biofilm-forming and biofilm-inhibited electrodes as measured by AFM. Statistical significance is indicated by an asterisk: ** for *p* < 0.01.

The root-mean-squared (RMS) roughness (*R*_q_) values obtained from AFM analysis are presented in Figure 5. While *S. aureus* exhibited ~24 nm, *S. epidermidis* biofilm showed ~16 nm, and this difference was statistically significant. Chatterjee et al. reported that RMS roughness values significantly increased upon biofilm formation, with 2-day-old *S. aureus* biofilm reaching 155 nm compared to 11 nm for the polycarbonate substrate control [45]. Their study suggests roughness is increased over time by biofilm formation. In our study *S. aureus* has the highest value but control itself is also a rough surface. Also, results suggest that *S. epidermidis* has the lowest roughness parameter that might be related to its compact biofilm.

## 4. Conclusions

In this study, we report on a simple and promising electrochemical impedance spectroscopy-based strategy for detecting bacterial biofilm formation using a low-cost two-electrode sensor. This EIS-based sensor was able to sensitively detect biofilm formed by *S. aureus* and *S. epidermidis*. While *R*_s_ values exhibited insignificant results, the *R*_ct_ values exhibited significant increase confirming biofilm presence. The control *R*_ct_ value increased by ~90 kΩ for *S. epidermidis* biofilm and by ~60 kΩ for *S. aureus* biofilms. The system is expected to work as a “biofilm-on-chip” for testing various agents preventing formation of biofilms or realizing degradation of the biofilms in the presence of antibiotics, bacteriophages, and other active bioreagents influencing the behavior of microbes. Here, preliminary attention was focused on AMO; this antibiotic treatment showed similar *R*_ct_ value to control, i.e., prevented formation of the biofilm. In addition, optical microscopy confirmed the presence of biofilm on the electrode surfaces. Atomic force micrographs and height profiles suggest that *S. aureus* preserved the individual shape but *S. epidermidis* showed an irregular profile and a compact, possibly caused by EPS. RMS roughness provided insight into the *S. aureus* biofilm being rougher than *S. epidermidis* biofilm. These findings demonstrate that the developed biosensor can detect a biofilm formation sensitively by evaluating *R*_ct_. Future research will further evaluate the efficacy of various antimicrobial compounds using the proposed methodology.

## Figures and Tables

**Table 1:** Fitting of the selected equivalent circuits to the experimental EIS scans for control and biofilm-modified electrodes. The Chi-squared (χ^2^, sum of squares of residuals from the fits) values characterize the tightness of individual fits. The values of fitted parameters are followed by their relative standard deviations (%, the parenthesised values). The units for individual parameters were kΩ for all R, nF for all capacitance values C, kσ for all W, and nT for Q values (based on the Equivalent Circuit Analysis module of the PSTrace software).

Equivalent Circuit	Control	Biofilm 1	Biofilm 2
*R*_s_(*R*_ct_*C*)	χ^2^ = 0.014	χ^2^ = 0.013	χ^2^ = 0.014
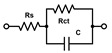	*R*_s_ = 1.04 (5.5) *R*_ct_ = 67.0 (4.1) *C* = 14.3 (4.3)	*R*_s_ = 0.996 (5.4) *R*_ct_ = 109 (4.5) *C* = 18.5 (4.1)	*R*_s_ = 0.995 (5.5) *R*_ct_ = 88.8 (4.3) *C* = 16.6 (4.2)
*R*_s_([*R*_ct_*W*]*C*)	χ^2^ = 0.0040	χ^2^ = 0.33	χ^2^ = 0.37
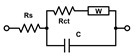	*R*_s_ = 1.03 (3.0) *R*_ct_ = 60.1 (3.0) *C* = 14.0 (2.4) *W* = 60.3 (16)	*R*_s_ = 0.93 (na) *R*_ct_ = 10^−6^ (inf) *C* = 6.7 (60) *W* = 477 (22)	*R*_s_ = 0.91 (na) *R*_ct_ = 10^−6^ (inf) *C* = 5.2 (87) *W* = 408 (22)
*R*_s_(*R*_1_*C*_1_)(*R*_ct_*C*)	χ^2^ = 0.0054	χ^2^ = 0.0054	χ^2^ = 0.0059
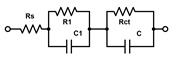	*R*_s_ = 1.04 (3.5) *R*_1_ = 21.9 (21) *C*_1_ = 1250 (38) *R*_ct_ = 60.4 (4.1) *C* = 14.3 (2.8)	*R*_s_ = 0.98 (3.4) *R*_1_ = 105 (4.6) *C*_1_ = 25.7 (11) *R*_ct_ = 8.32 (51) *C* = 47.9 (3.6)	*R*_s_ = 0.99 (3.6) *R*_1_ = 41.8 (23) *C*_1_ = 127 (54) *R*_ct_ = 56.0 (18) *C* = 18 (7.7)
*R*_s_([*R*_ct_*W*]*Q*)	χ^2^ = 0.00030	χ^2^ = 0.00010	χ^2^ = 0.00050
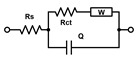 *(Randles)*	*R*_s_ = 0.99 (0.90) *R*_ct_ = 63.9 (0.90) *W* = 47.8 (5.9) *Q* = 21.8 (3.1) *n* = 0.95 (0.3)	*R*_s_ = 0.94 (0.60) *R*_ct_ = 108 (0.70) *W* = 49.9 (6.6) *Q* = 30.4 (1.9) *n* = 0.94 (0.20)	*R*_s_ = 0.94 (1.2) *R*_ct_ = 86.4 (1.4) *W* = 52.5 (10) *Q* = 26.3 (3.9) *n* = 0.94 (0.40)

Abbreviations: na not available; inf infinity.

**Table 2:** The analysis of the coverage area and maximum profile height (Pt) for AFM scans of different biofilm surfaces, including the effect of amoxicillin. The mean values ± standard deviations are shown.

Sample	Coverage Area (µm^2^)	Max. Profile Height (µm)
*S. epidermidis* 10 min	18.4 ± 7.7	0.74 ± 0.068
*S. epidermidis* 1 h	88 ± 15	0.66 ± 0.13
*S. epidermidis* with AMO	0	0.24 ± 0.036
*S. aureus* 10 min	72 ± 23	0.86 ± 0.11
*S. aureus* 1 h	80 ± 24	1.0 ± 0.051
*S. aureus* with AMO	1.3 ± 1.8	0.46 ± 0.18

## Data Availability

Data is contained within the article.

## References

[1] Mahto K.U., Vandana, Priyadarshanee M., Samantaray D.P., Das S. (2022). Bacterial Biofilm and Extracellular Polymeric Substances in the Treatment of Environmental Pollutants: Beyond the Protective Role in Survivability. J. Clean. Prod..

[2] Wang Y., Bian Z., Wang Y. (2022). Biofilm Formation and Inhibition Mediated by Bacterial Quorum Sensing. Appl. Microbiol. Biotechnol..

[3] Karygianni L., Ren Z., Koo H., Thurnheer T. (2020). Biofilm Matrixome: Extracellular Components in Structured Microbial Communities. Trends Microbiol..

[4] Guzmán-Soto I., McTiernan C., Gonzalez-Gomez M., Ross A., Gupta K., Suuronen E.J., Mah T.-F., Griffith M., Alarcon E.I. (2021). Mimicking Biofilm Formation and Development: Recent Progress in in vitro and in vivo Biofilm Models. iScience.

[5] Kreve S., Reis A.C.D. (2021). Bacterial Adhesion to Biomaterials: What Regulates This Attachment? A Review. Jpn. Dent. Sci. Rev..

[6] Wang C., Hou J., van der Mei H.C., Busscher H.J., Ren Y. (2019). Emergent Properties in *Streptococcus mutans* Biofilms Are Controlled through Adhesion Force Sensing by Initial Colonizers. mBio.

[7] Palmer J., Flint S., Brooks J. (2007). Bacterial Cell Attachment, the Beginning of a Biofilm. J. Ind. Microbiol. Biotechnol..

[8] Theis T.J., Daubert T.A., Kluthe K.E., Brodd K.L., Nuxoll A.S. (2023). *Staphylococcus aureus* Persisters Are Associated with Reduced Clearance in a Catheter-Associated Biofilm Infection. Front. Cell Infect. Microbiol..

[9] Conlon B.P. (2014). *Staphylococcus aureus* Chronic and Relapsing Infections: Evidence of a Role for Persister Cells: An Investigation of Persister Cells, Their Formation and Their Role in *S. aureus* Disease. BioEssays.

[10] Lewis K. (2007). Persister Cells, Dormancy and Infectious Disease. Nat. Rev. Microbiol..

[11] Zhao A., Sun J., Liu Y. (2023). Understanding Bacterial Biofilms: From Definition to Treatment Strategies. Front. Cell Infect. Microbiol..

[12] Rather M.A., Gupta K., Mandal M. (2021). Microbial Biofilm: Formation, Architecture, Antibiotic Resistance, and Control Strategies. Braz. J. Microbiol..

[13] Del Pozo J.L. (2018). Biofilm-Related Disease. Expert. Rev. Anti Infect. Ther..

[14] Severn M.M., Horswill A.R. (2023). *Staphylococcus epidermidis* and Its Dual Lifestyle in Skin Health and Infection. Nat. Rev. Microbiol..

[15] Idrees M., Sawant S., Karodia N., Rahman A. (2021). *Staphylococcus aureus* Biofilm: Morphology, Genetics, Pathogenesis and Treatment Strategies. Int. J. Environ. Res. Public Health.

[16] Diepoltová A., Konečná K., Janďourek O., Nachtigal P. (2021). Study of the Impact of Cultivation Conditions and Peg Surface Modification on the in vitro Biofilm Formation of *Staphylococcus aureus* and *Staphylococcus epidermidis* in a System Analogous to the Calgary Biofilm Device. J. Med. Microbiol..

[17] Arciola C.R., Campoccia D., Montanaro L. (2002). Detection of Biofilm-Forming Strains of *Staphylococcus epidermidis* and *S. aureus*. Expert. Rev. Mol. Diagn..

[18] Khatoon Z., McTiernan C.D., Suuronen E.J., Mah T.-F., Alarcon E.I., Alarcon Bacterial E.I. (2018). Bacterial Biofilm Formation on Implantable Devices and Approaches to Its Treatment and Prevention. Heliyon.

[19] Li G., Wu Y., Li Y., Hong Y., Zhao X., Reyes P.I., Lu Y. (2020). Early Stage Detection of *Staphylococcus epidermidis* Biofilm Formation Using MgZnO Dual-Gate TFT Biosensor. Biosens. Bioelectron..

[20] Guła G., Szymanowska P., Piasecki T., Góras S., Gotszalk T., Drulis-Kawa Z. (2020). The Application of Impedance Spectroscopy for *Pseudomonas* Biofilm Monitoring during Phage Infection. Viruses.

[21] Achinas S., Yska S.K., Charalampogiannis N., Krooneman J., Euverink G.J.W. (2020). A Technological Understanding of Biofilm Detection Techniques: A Review. Materials.

[22] Ameer S., Ibrahim H., Yaseen M.U., Kulsoom F., Cinti S., Sher M. (2023). Electrochemical Impedance Spectroscopy-Based Sensing of Biofilms: A Comprehensive Review. Biosensors.

[23] Nag M., Lahiri D. (2021). Analytical Methodologies for Biofilm Research.

[24] Kretzschmar J., Harnisch F. (2021). Electrochemical Impedance Spectroscopy on Biofilm Electrodes—Conclusive or Euphonious?. Curr. Opin. Electrochem..

[25] Romero M.C., Méndez-Tovar M. (2023). Impedance Analysis for the Study of Biofilm Formation on Electrodes: An Overview. J. Mex. Chem. Soc..

[26] Xu W., Ceylan Koydemir H. (2022). Non-Invasive Biomedical Sensors for Early Detection and Monitoring of Bacterial Biofilm Growth at the Point of Care. Lab. Chip.

[27] Swami P., Anand S., Holani A., Gupta S. (2024). Impedance Spectroscopy for Bacterial Cell Monitoring, Analysis, and Antibiotic Susceptibility Testing. Langmuir.

[28] Paredes J., Becerro S., Arana S. (2014). Comparison of Real Time Impedance Monitoring of Bacterial Biofilm Cultures in Different Experimental Setups Mimicking Real Field Environments. Sens. Actuators B Chem..

[29] McGlennen M., Dieser M., Foreman C.M., Warnat S. (2023). Monitoring Biofilm Growth and Dispersal in Real-Time with Impedance Biosensors. J. Ind. Microbiol. Biotechnol..

[30] Ward A.C., Hannah A.J., Kendrick S.L., Tucker N.P., MacGregor G., Connolly P. (2018). Identification and Characterisation of *Staphylococcus aureus* on Low Cost Screen Printed Carbon Electrodes Using Impedance Spectroscopy. Biosens. Bioelectron..

[31] Kim T., Kang J., Lee J.H., Yoon J. (2011). Influence of Attached Bacteria and Biofilm on Double-Layer Capacitance during Biofilm Monitoring by Electrochemical Impedance Spectroscopy. Water Res..

[32] Lacina K., Věžník J., Sopoušek J., Farka Z., Lacinová V., Skládal P. (2023). Concentration and Diffusion of the Redox Probe as Key Parameters for Label-Free Impedimetric Immunosensing. Bioelectrochemistry.

[33] Obořilová R., Šimečková H., Pastucha M., Klimovič Š., Víšová I., Přibyl J., Vaisocherová-Lísalová H., Pantůček R., Skládal P., Mašlaňová I. (2021). Atomic Force Microscopy and Surface Plasmon Resonan Ce for Real-Time Single-Cell Monitoring of Bacteriophage-Mediated Lysis of Bacteria. Nanoscale.

[34] Sopoušek J., Věžník J., Houser J., Skládal P., Lacina K. (2021). Crucial Factors Governing the Electrochemical Impedance on Protein-Modified Surfaces. Electrochim. Acta.

[35] Standar K., Kreikemeyer B., Redanz S., Münter W.L., Laue M., Podbielski A. (2010). Setup of an in vitro Test System for Basic Studies on Biofilm Behavior of Mixed-Species Cultures with Dental and Periodontal Pathogens. PLoS ONE.

[36] Obořilová R., Kučerová E., Botka T., Vaisocherová-Lísalová H., Skládal P., Farka Z. (2025). Piezoelectric Biosensor with Dissipation Monitoring Enables the Analysis of Bacterial Lytic Agent Activity. Sci. Rep..

[37] Deng M., Wang Y., Chen G., Liu J., Wang Z., Xu H. (2021). Poly-L-Lysine-Functionalized Magnetic Beads Combined with Polymerase Chain Reaction for the Detection of *Staphylococcus aureus* and *Escherichia coli* O157:H7 in Milk. J. Dairy Sci..

[38] Marka S., Zografaki M.E., Papaioannou G.M., Mavrikou S., Flemetakis E., Kintzios S. (2023). Impedance In vitro Assessment for the Detection of *Salmonella typhimurium* Infection in Intestinal Human Cancer Cells. Chemosensors.

[39] Kim B.R., Bae Y.M., Lee S.Y. (2016). Effect of Environmental Conditions on Biofilm Formation and Related Characteristics of *Staphylococcus aureus*. J. Food Saf..

[40] Oliver L.M., Dunlop P.S.M., Byrne J.A., Blair I.S., Boyle M., Mcguigan K.G., Mcadams E.T. An Impedimetric Sensor for Monitoring the Growth of *Staphylococcus epidermidis*. Proceedings of the 2006 International Conference of the IEEE Engineering in Medicine and Biology Society.

[41] Lofrumento C., Arci F., Carlesi S., Ricci M., Castellucci E., Becucci M. (2015). Safranin-O Dye in the Ground State. A Study by Density Functional Theory, Raman, SERS and Infrared Spectroscopy. Spectrochim. Acta A Mol. Biomol. Spectrosc..

[42] Ommen P., Zobek N., Meyer R.L. (2017). Quantification of Biofilm Biomass by Staining: Non-Toxic Safranin Can Replace the Popular Crystal Violet. J. Microbiol. Methods.

[43] James S.A., Powell L.C., Wright C.J. (2016). Atomic Force Microscopy of Biofilms—Imaging, Interactions, and Mechanics. Microbial Biofilms—Importance and Applications.

[44] Tollersrud T., Berge T., Andersen S.R., Lund A. (2001). Imaging the Surface of *Staphylococcus aureus* by Atomic Force Microscopy. APMIS.

[45] Chatterjee S., Biswas N., Datta A., Dey R., Maiti P. (2014). Atomic Force Microscopy in Biofilm Study. Microscopy.

